# Subcutaneous Dirofilariasis of the Lateral Cervical Region: A Case Report

**DOI:** 10.7759/cureus.92826

**Published:** 2025-09-21

**Authors:** Dimitrios Nikas, Andreas Koumenis, Theodoros Piperos, Theodoros Mariolis-Sapsakos

**Affiliations:** 1 Laboratory of Anatomy, Advanced Anatomical Applications, Artificial Intelligence, and Experimental Surgical Research (AAAAAIES Lab), Department of Nursing, National and Kapodistrian University of Athens, Athens, GRC

**Keywords:** cervical dirofilariasis, dirofilaria repens, parasitic infection, subcutaneous nodule, zoonosis and public health

## Abstract

*Dirofilaria repens* (*D. repens*)is an emerging zoonotic filarial parasite, increasingly implicated in human subcutaneous infections. While commonly presenting as solitary nodules in the ocular or facial regions, involvement of atypical anatomical sites remains rare and diagnostically challenging.

We report a case of subcutaneous cervical *D. repens* infection in a 50-year-old woman from Greece with no history of travel or contact with animals. The patient presented with a firm, non-tender nodule over the sternocleidomastoid muscle. Ultrasound revealed a hypoechoic lesion with soft tissue involvement. Surgical excision revealed a cystic cavity containing a filamentous structure. Histopathological analysis demonstrated nematode larval sections with eosinophilic infiltration, and polymerase chain reaction (PCR) confirmed the presence of *D. repens*. The postoperative course was uneventful, and no antiparasitic therapy was required.

This case comes to enrich the reported cases of atypical *D. repens* presentations, highlighting the need for clinical awareness even among patients without classic exposure risks. Given its nonspecific clinical features, *D. repens* infection should be considered in the differential diagnosis of subcutaneous nodules, particularly when initial imaging and clinical evaluation do not align with neoplastic or inflammatory etiologies. Surgical excision remains both diagnostic and curative in most cases.* *Informed consent was obtained from the patient for publication.

## Introduction

Human dirofilariasis is an emerging zoonotic infection caused by filarial nematodes of the genus *Dirofilaria*, most commonly *Dirofilaria repens* (*D. repens*). Domestic dogs and other canids serve as the primary reservoir hosts, while humans are incidental, dead-end hosts in whom the parasite’s development typically halts at immature stages. Transmission occurs via mosquito vectors, and clinical manifestations are often subtle and nonspecific, most commonly presenting as solitary subcutaneous or ocular nodules [1,2]. In recent decades, the incidence of human *D. repens* infections has increased significantly, driven by climatic changes favorable to mosquito proliferation, international pet travel, and cross-border animal adoption [3,4]. Despite this rising trend, many clinicians remain unfamiliar with the disease, especially in non-endemic areas or when it manifests in unusual anatomical sites. These atypical presentations often mimic neoplastic or inflammatory conditions, complicating diagnosis and delaying treatment [5,6]. Here, we present a rare case of subcutaneous cervical *D. repens* infection in a middle-aged woman from Greece with no travel or pet exposure. A brief literature review follows, highlighting the challenges associated with atypical presentations.

## Case presentation

**Figure 1:**
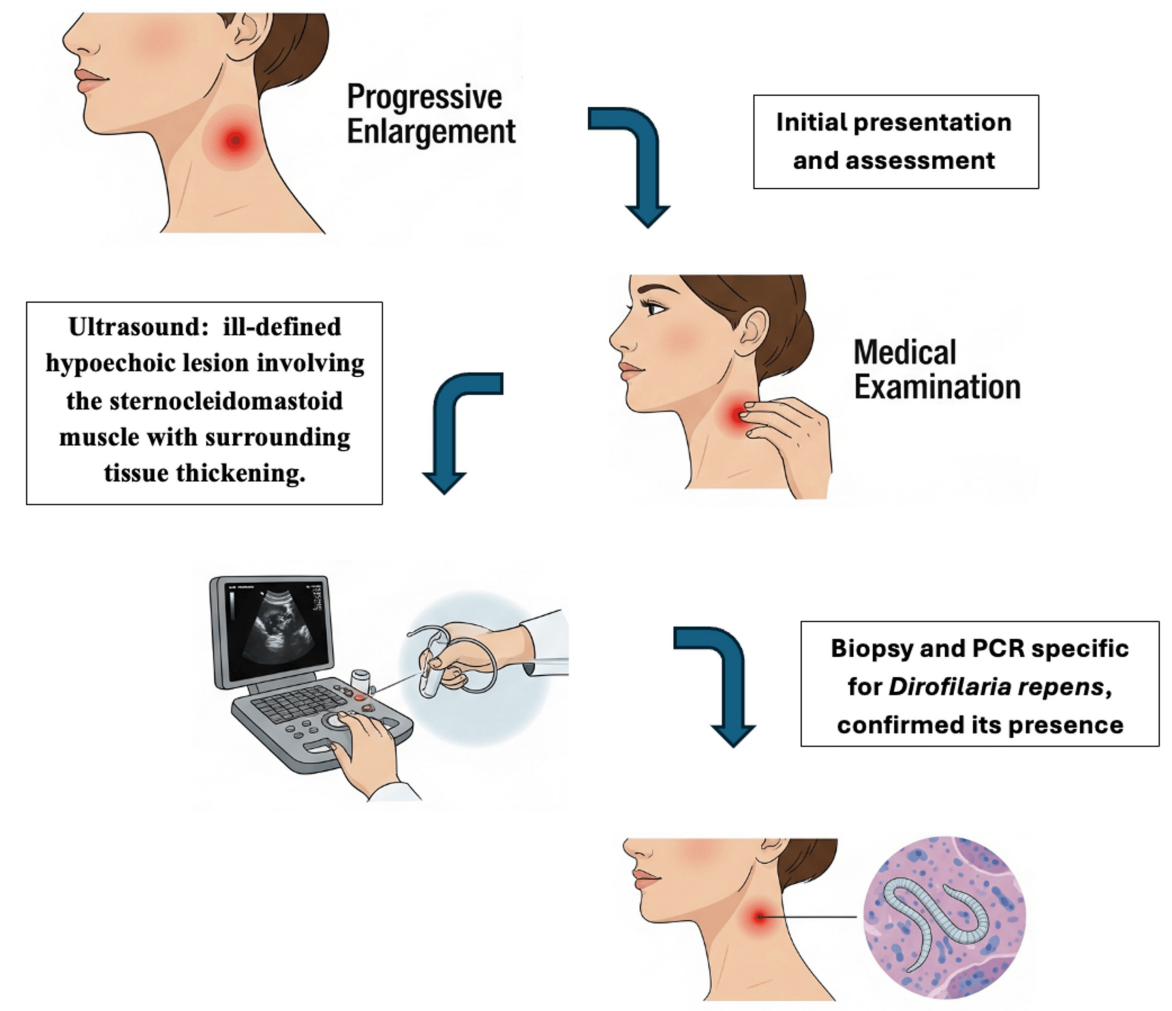
Schematic representation of diagnostic workup and clinical timeline The components of this figure were created specifically for this manuscript using AI-generated illustrations produced with Nano Banana, Gemini’s image generator (Google Inc., Mountain View, CA), under the authors’ direction. PCR: polymerase chain reaction

A 50-year-old Greek woman, residing in the northwestern suburbs of Attica, Greece, presented in early July of 2024 at our outpatient clinic with a solitary, firm, subcutaneous nodule measuring 2 × 2.5 cm on the lateral aspect of her cervical region, superior to the sternocleidomastoid muscle. The patient reported the lesion had begun as a non-tender lump that progressively enlarged and subsequently became pruritic. She denied any pain or tenderness associated with the nodule. She reported no relevant prior medical history and presented in good general condition and was afebrile. Initial differential diagnoses included benign lesions such as lipoma and epidermoid cyst, neoplastic conditions such as sarcoma or metastatic lymphadenopathy, and inflammatory/infectious causes, including tuberculous lymphadenitis and bacterial abscess. The nodule's location resulted in a subjective restriction of neck movement. Notably, the patient reported no history of travel outside of Greece or contact with pets. Initial physical examination revealed no other similar lesions. Routine laboratory investigations yielded results within normal limits. Ultrasound imaging of the neck demonstrated an ill-defined hypoechoic lesion in the lateral cervical region, appearing to involve the sternocleidomastoid muscle with surrounding tissue thickening but no vascular invasion, which argued against malignancy. The absence of pain, systemic symptoms, or fluctuance also reduced the likelihood of bacterial abscess. These features prompted a surgical biopsy for definitive diagnosis. Based on the presenting signs and the differential diagnosis of possible infectious, neoplastic, or inflammatory etiology, the patient consented to proceed with biopsy of the nodule. Consequently, an excisional biopsy was performed under local anesthesia (Figure 1).

**Figure 2:**
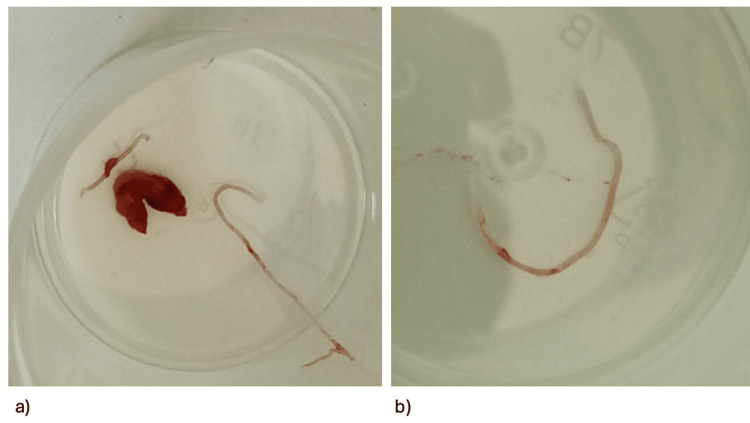
a) The cystic cavity was approximately 19 mm; b) the filamentous structure was approximately 12 mm in length and 1 mm in thickness.

Gross examination of the excised specimen revealed a cystic cavity with a maximum diameter of approximately 19 mm. Upon incision, a filamentous structure, approximately 12 mm in length and 1 mm in thickness, was identified within the cyst and submitted to the microbiology department for identification (Figure 2).

Peripheral blood smear for microfilariae was negative. Histopathological analysis of the cyst wall revealed multiple cross-sections of a nematode larva accompanied by eosinophilic inflammatory infiltration. The nematode had a thick, multilayered cuticle bearing longitudinal ridges, a well-developed muscle layer, and a central intestine, morphological hallmarks consistent with *D. repens*. Microbiological analysis, including polymerase chain reaction (PCR) specific for *D. repens*, confirmed the presence of this nematode. Given the localized nature of the infection and the absence of further signs or symptoms, no antiparasitic treatment was deemed necessary following complete surgical excision. The postoperative course was uneventful, with complete wound healing and suture removal on postoperative day seven. The patient was followed up clinically at one, three, and six months postoperatively, with no recurrence or systemic symptoms. Blood smears remained negative for microfilariae. 

## Discussion

Human dirofilariasis is a zoonotic infection caused by filarial nematodes of the genus *Dirofilaria*. While several *Dirofilaria *species, such as *Dirofilaria immitis*, *D. repens*, and* Dirofilaria tenuis,* can infect humans, *D. repens* is recognized as the predominant causative agent of human dirofilariasis across Europe, Asia, and Africa [1]. The primary reservoir hosts for *D. repens* are domestic and wild canids, particularly dogs [2]. Humans are typically considered aberrant or accidental hosts, meaning the parasite's life cycle within them is usually incomplete [3]. The lack of travel or pet contact in our case strongly suggests autochthonous transmission, likely mediated by local mosquito vectors. This supports recent findings that *D. repens* transmission is no longer confined to classical endemic regions but is expanding across Europe under changing ecological conditions. These epidemiological trends indicate a significant increase in reported clinical cases of dirofilariasis in both animals and humans [3]. This rise may be attributed to several interconnected factors. Changes in climatic conditions, including elevated temperatures, increased relative humidity, and higher rainfall, create more favorable environments for mosquito vectors and facilitate the development of larval stages within these vectors [4]. This environmental shift directly contributes to the expansion of the geographical range of competent mosquito species [5]. Concurrently, anthropogenic factors such as increased international travel, the movement of infected pets, and the adoption of dogs from endemic areas play a substantial role in disseminating the parasite into previously non-endemic territories [4]. However, cervical presentations of subcutaneous *D. repens* remain rare in Europe, with only a few cases reported to date. The reported manifestations of *D. repens* in Greece are also scarce, underscoring the need for heightened awareness of atypical site presentations. Human *D. repens* infection typically manifests as subcutaneous or cutaneous nodules, which represent the host's inflammatory response to the presence of pre-adult or adult parasites [5]. These nodules are generally solitary, well-defined, firm, and elastic, often growing gradually over several weeks or months [2]. Cases show the highest incidence of subcutaneous nodules in individuals aged 40 to 49 years, though infections occur across all age groups, including children [6]. Women appear to be more susceptible to subcutaneous dirofilariasis than men, with a reported ratio of 55.4% versus 44.6% [6]. Common anatomical sites for these subcutaneous lesions include the face, periorbital region, limbs (upper arms, thighs, and lower legs), chest wall, and abdominal wall [7]. Ocular dirofilariasis, characterized by subconjunctival, intravitreal, or intraorbital infections, is another common presentation, leading to inflammatory reactions, visual disturbances, and potential vision loss [5]. In the literature, there are scattered cases of rare anatomical sites’ presentations of human *D. repens*. Chan et al. (2013) presented a case of a 47-year-old man with a lower anterolateral non-tender neck swelling, which was diagnosed as *D. repens* infection [8]. This was considered an unusual occurrence and, as mentioned by the author at the time of publication, the first such report in Europe [8]. Choudhury et al. (2023) presented another case, this time in Asia, of a slowly growing, firm swelling in the lateral aspect of the patient’s left neck, which was subsequently confirmed as a *Dirofilaria *infection [7]. In addition to cervical involvement, Schneider et al. (2025) reported a case involving an 18-year-old male patient who presented with a painless subcutaneous swelling on the left cheek. The diagnosis of subcutaneous dirofilariasis was confirmed post-surgical excision using nematode-specific PCR targeting the 12S rRNA gene, followed by sequencing analysis [4].

Another rare manifestation was published by Ciuca et al. (2025) in Italy. A 67-year-old man presented with an oval-shaped subcutaneous mass in the left frontal region, initially presumed to be a subcutaneous cyst. However, a live worm was discovered during surgical excision, and histological analysis confirmed the presence of *D. repens* [2].

Also, Ciuca, in the same manuscript, reported the case of a 33-year-old man with complaints of pruritus and swelling of the left upper eyelid, which progressed to form a lump. Surgical excision of the nodule, followed by a histology report, confirmed the presence of *D. repens* [2]. Another rare instance of a left inguinal mass that was progressing in size in a 41-year-old woman was reported by Schatz et al. (2025). The mass was surgically removed, and the diagnosis of dirofilariasis by *D. repens* was set [5]. A 56-year-old woman with bulging and pain in the right inguinal lymph node is also reported by Ermakova et al. (2020), where, after the surgical excision, fragments of the helminth were found during histology and identified with PCR as being *D. repens* [9]. Leccia et al. (2012) reported two cases of male genital dirofilariasis. A 29-year-old man with a nodule in the left epididymis and a 66-year-old man with a firm and movable nodule attached to the spermatic cord. Both cases represent another challenging atypical presentation of *D. repens* infection [10]. Breast dirofilariasis was reported by Prasad et al. (2019) with a breast lump that mimics a malignant tumor, thus proving to be a diagnostic challenge [11]. Lastly, an unusual case in a patient who had pleural and subcutaneous (upper trunk and axillary regions) *D. repens* with clinical manifestation was reported by Biasizzo et al. (2022). A video-assisted thoracoscopic surgical excision of the pleural lesion was performed, with PCR from the tissue confirming the presence of *D. repens* [12]. This brief literature review of the atypical localizations of *D. repens* presentations underscores the diagnostic challenge it poses. This diagnostic ambiguity often leads to unnecessary invasive investigations, extensive workups, and surgical procedures before the correct parasitic etiology is established [3]. The non-specific nature of symptoms, such as localized swelling, pain, or itching, further contributes to this diagnostic difficulty [7]. Traditionally, dirofilariasis was diagnosed through morphological examination of parasite samples, such as microfilariae and adult worms obtained from surgical excision or biopsy. This remains the definitive method for diagnosing dirofilariasis, offering conclusive evidence and a comprehensive understanding of the disease pathology. However, identification can be challenging due to worm decomposition, similar cuticular morphologies across Dirofilaria species, and variability in key features like cuticular ridge size and number, prompting the shift towards more advanced molecular diagnostic techniques [1]. These techniques include identifying and examining genetic material from the biopsied site directly, providing significant benefits compared to conventional morphological approaches. Polymerase chain reaction (PCR) has become widely used for diagnosing dirofilariasis because of its superior sensitivity and specificity [1]. Peripheral blood smears and serology, while a common and cost-effective method, are of limited diagnostic value in human *D. repens* infections, due to the typically absent or low-level microfilaremia leading to false-negative results [4]. Histopathology allows visualization of nematode morphology within tissue, while PCR targeting species-specific gene regions offers high sensitivity and specificity, reducing misidentification risk. For these reasons, histology and PCR were prioritized in our diagnostic approach.

Ultrasound can reveal encapsulated masses, hypoechoic lesions, and sometimes even the characteristic "filarial dance sign" (moving tubular structures) [13]. However, imaging may not detect early-stage infections, differentiate *Dirofilaria* from other conditions, or provide definitive species identification. The mainstream treatment for localized human subcutaneous *D. repens* infection is the surgical removal of the nodule containing the parasite [7]. This approach is almost always curative, especially when only a single parasite is present and the infection is localized [7]. In many reported cases, including those with head and neck involvement, complete recovery is achieved without the need for further treatment [14].

There are no standardized treatment guidelines for dirofilariasis or microfilaremia, so therapeutic choices are typically left to the discretion of the treating physician. Surgical removal remains the main approach, often supplemented by adjuvant therapies like ivermectin, albendazole, or diethylcarbamazine. Additionally, doxycycline has been employed to target *Wolbachia *endosymbionts associated with the parasite [15].

This case report is limited by the absence of serological testing, which may have contributed additional epidemiological information despite its low sensitivity in human *D. repens* infections. In addition, this single case cannot establish broader epidemiological trends; larger-scale surveillance studies are needed to clarify the prevalence of atypical localizations and transmission dynamics in Greece.

## Conclusions

The presented case of subcutaneous human *D. repens* infection in the cervical region of a 50-year-old female patient contributes to the growing body of literature on atypical clinical presentations of this emerging zoonosis. While subcutaneous nodules are a common manifestation, the specific localization in the neck is rare and often leads to diagnostic challenges, including misdiagnosis as neoplastic lesions, as highlighted by previous reports. Surgical removal of the parasitic cyst and the worm itself, as in this case, is typically sufficient for recovery. Given the increasing global incidence of *D. repens* infections, driven by climate change and human activities, a high index of suspicion for dirofilariasis is paramount for clinicians, especially when encountering subcutaneous nodules in unusual anatomical locations, particularly for unexplained cervical or facial swellings. Ultrasound-guided excision and histopathological examination should be prioritized, with molecular confirmation where available.
